# Dehydrated Human Amnion-Chorion Membrane Extracts Can Ameliorate Interstitial Cystitis in Rats by Down-Regulating Inflammatory Cytokines and Protein Coding Genes: A Preclinical Study

**DOI:** 10.3390/life12111693

**Published:** 2022-10-25

**Authors:** Che-Hsueh Yang, Min-Che Tung, Yi-Sheng Lin, Chao-Yu Hsu, Ivan Akhrymuk, Kok-Tong Tan, Yen-Chuan Ou, Chi-Chien Lin

**Affiliations:** 1 Division of Urology, Department of Surgery, Tungs’ Taichung MetroHarbor Hospital, Taichung 435, Taiwan; 2 PhD Program in Translational Medicine, Rong Hsing Research Center for Transitional Medicine, National Chung Hsing University, Taichung 402, Taiwan; 3 Department of Biomedical Science and Pathobiology, Virginia-Maryland College of Veterinary Medicine, Virginia Polytechnic Institute and State University, Blacksburg, VA 24060, USA; 4 Division of General Surgery, Department of Surgery, Tungs’ Taichung MetroHarbor Hospital, Taichung 435, Taiwan; 5 Institute of Biomedical Science, The iEGG and Animal Biotechnology Center, National Chung Hsing University, Taichung 402, Taiwan; 6 Department of Biotechnology, Asia University, Taichung 413, Taiwan; 7 Department of Medical Research, China Medical University Hospital, China Medical University, Taichung 406, Taiwan; 8 Department of Medical Research, Taichung Veterans General Hospital, Taichung 407, Taiwan; 9 Department of Pharmacology, College of Medicine, Kaohsiung Medical University, Kaohsiung 807, Taiwan

**Keywords:** animal, anti-infective agents/therapeutic use, amnion/transplantation, cystitis, interstitial/therapy, chorion transplantation

## Abstract

The study aimed to investigate the therapeutic impact of intravesical instillation of dehydrated human amnion-chorion membrane (HACM) extracts based on the primary pathological feature of interstitial cystitis (IC). We divided 15 female Sprague-Dawley rats into three groups: sham control, IC, and treatment group. IC was induced by 400-µL lipopolysaccharide (1 µg/µL), and it was replaced with normal saline in the sham control group. After IC induction, 300 µL dehydrated HACM extracts (3 mg/kg) were instilled into rats’ urinary bladder weekly for 3 weeks. General histology, inflammatory cytokines, NF-κB, oxidative markers, and western blots results were examined. The urothelial denudation, mast-cell infiltration, and tissues fibrosis were all ameliorated. The elevated TNF-α, IL-1β, IL-6, IL-8, and NF-κB were all down-regulated by dehydrated HACM extracts (*p* < 0.05). For reactive oxygen species, increased malondialdehyde, decreased superoxide dismutase, and decreased glutathione peroxidase were all reversed (*p* < 0.05). In apoptosis of IC, elevated Bax and suppressed Bcl-2 were improved (*p* < 0.05) after instillation. In fibrosis, dysregulated TGFβ/R-Smads/Snail was corrected by the instillation of dehydrated HACM (*p* < 0.05). In conclusion, dehydrated HACM extracts could be a powerful remedy in treating IC by reconstructing the damaged urothelium, reducing mast-cell infiltration and inflammatory reactions, and ameliorating fibrotic changes.

## 1. Introduction

According to American Urological Association (AUA) recommendations [1,2,3], interstitial cystitis (IC) is diagnosed when lower urinary tract symptoms are accompanied by consistent painful sensations for more than 6 weeks without any apparent explanations, such as a urinary tract infection. However, several different descriptions may be found in different guidelines. For example, the European Society for the Study of Interstitial Cystitis (ESSIC) adopted the term bladder pain syndrome to replace the nomenclature of IC in 2009. ESSIC emphasized excluding the possibilities of confounding conditions, other than establishing the specific symptoms or signs for it [4]. Thus, the fundamental framework in diagnosing IC is to exclude other possible diseases or to ensure there are other coexisting diseases. The clinical diagnosis of IC is challenging for urologists because there are no symptoms related explicitly to IC, and the clinical responses to tests do not necessarily indicate the presence of IC. Furthermore, there are no laboratory biomarkers suggested by the AUA and ESSIC guidelines to assist the urologists in IC diagnosis, making the diagnosis more ambiguous and complex [3,4]. Consequently, patients often suffer from delayed diagnosis or misdiagnosis. In the current literature, the inconsistent and ambiguous diagnostic criteria used to determine the prevalence of IC vary considerably [5,6,7,8,9].

The AUA and ESSIC guidelines only provide diagnostic principles for IC diagnosis; nevertheless, pathophysiological alterations observed under the microscope may provide clues on how these clinical symptoms manifest in patients with IC. The urothelium constitutes the protective fence against the irritative components in urine, such as potassium. The irritative components might infiltrate the underlying sensory receptors once the urothelial denudation exists, leading to the manifestation of the symptoms [10]. For example, the urothelial denudation of IC could increase the potassium permeability, resulting in mast-cell infiltration. The former leads to tissues injuries by depolarizing nerves and muscles [11], and the latter causes vascular remodeling by secreting excessive vasoactive and pro-inflammatory mediators [12]. In the chronic inflammatory process, mast cell gathering is another pivotal change in the pathophysiology of IC. In mediators secreted by mast cells, cytokines, such as IL-6, IL-1β, and TNF-α, cause inflammation, pain, and vasodilation. Moreover, they also produce chemokines, such as IL-8, resulting in chemoattraction responsible for the migration of immune cells, phagocytosis, and angiogenesis [13]. Consequently, dysregulated mast cells damage urinary bladder (UB) tissues, causing IC to deteriorate. In conclusion, the two principal features of urothelial denudation and chronic inflammation may enhance the neural sensitivity of UB [14] and deteriorate the clinical symptoms. One of the main goals of developing treatments for IC is to improve the pathophysiological changes mentioned above.

Currently, IC treatment options comprise oral medication and intravesical instillation or injection. In oral medication, the treatment goals focus on replenishing the glycosaminoglycan (GAG) layer, suppressing the deteriorating effects of mast cells by antihistamine drugs, relaxing UB, and reducing other irritating symptoms by antidepressants/painkillers/nonsteroidal anti-inflammatory drugs. Intravesical therapies share the same treatment goals as oral medication, and some other substances, such as botulinum toxin, dimethyl sulfoxide and hyaluronic acid (HA) are utilized in more deteriorating conditions refractory to oral medication. In some cases, intravesical steroid injections may be performed to control the progression of IC. In various options of intravesical instillation, HA and chondroitin sulfate (CS) were deemed as therapies of significant value [15]. However, although HA could significantly improve the symptoms of IC and the capacity of UB, its treatment effect might not persist for long duration [16]. As for CS, there was no significant global response assessment in the randomized controlled double-blinded trials [15], and its treatment effect on relieving painful UB was established by the synchronous use of HA [17].

Exploring a new intravesical agent with a better treatment effect and longer duration is still necessary. Recently, from observing the process of urothelial healing after surgeries, innate stem cells coexisted with CK5+/CK14+ basal cells in UB [18]. Therefore, a better treatment effect might be achieved by adding abundant exogenous growth factors. Since human amnion-chorion membrane extracts (HACM) contain several growth factors, they may help patients to repair damaged urothelium and reduce inflammation. However, although there were many instilled materials discussed in the recent literatures [15], dehydrated HACM extracts were never explored. In addition to the instilled materials, the selection of IC models was important. Furthermore, the rats’ IC model induced by lipopolysaccharide (LPS) was similar to human IC in terms of mast cell infiltration, urothelial histological changes, leukocytes’ recruitments, and production of inflammatory cytokines and chemokines [19,20]. In this preclinical study, we conducted the first animal experiment investigating the efficacy of dehydrated HACM extracts on LPS-induced UB inflammation in rats. Our primary objective was to assess the influence of dehydrated HACM extracts on the gross histological changes of the LPS-induced IC model. The secondary objective was to evaluate the impacts of dehydrated HACM extracts on the inflammatory cytokines, protein-encoding genes in fibrotic pathways, and apoptosis-related proteins of the LPS-induced IC model.

## 2. Materials and Methods

### 2.1. Animals

Adult female Sprague-Dawley (SD) rats (Japan SLC, Hamamatsu, Japan) weighing 200–300 g were used in this study. The SD rats were housed in a room at 22–24 °C and 50%–60% relative humidity with an alternating 12 h light/12 h dark cycle. Food and water were available ad libitum. All experimental procedures on rats were approved by the Committee of Animal Experiments in National Chung Hsing University (Taichung, Taiwan) and were performed in accordance with the Guide for the Care and Use of Laboratory Animals of the National Institutes of Health (Bethesda, MD, USA).

### 2.2. Rats Model Establishment

A total of 15 female SD rats were divided into 3 groups: sham control, IC, and treatment groups. In the sham control group, the UB was instilled with sterilized saline to imitate the distended changes of UB. In the IC group, LPS was applied to establish an IC model in rats. In the treatment group, the suspension of dehydrated HACM extracts was prepared according to the package insert and instilled into UB with LPS-induced IC immediately after preparation. Suspension of dehydrated HACM extracts was instilled at 48 h after IC induction to examine the treatment effects of dehydrated HACM extracts.

Rats were anesthetized by intramuscular injection of 16 mg/kg of xylazine (Rompun; Bayer Korea Corp, Seoul, Korea) and 0.04 mg/kg of zolazepam/tiletamine (Zoletil; Virbac, Carros, France) before establishing the access to UB from outside [20]. After adequate anesthetizations, an abdominal incision was made to expose the UB, followed by a minor incision at the UB dome. Next, one of the ends of a polyethylene-50 tube (Natsume Seisakusho Co., Tokyo, Japan) was placed inside the UB, and the UB was closed to be water-tight [18,19]. The contralateral end of the polyethylene tube was passed through the subcutaneous layer of the left flank area and pulled out at the posterior neck area. After confirming that the tube was unobstructed and that urine was draining well without leaking through the UB incision, the polyethylene-50 tube was secured, and the abdominal incision was closed. An intramuscular injection of 15 mg/kg of cefprozil was given to avoid procedure-related infections. Thereafter, the rats were monitored in warm cages until they fully awakened [21].

### 2.3. Induction of LPS-Induced Cystitis and Dehydrated HACM Extracts Administration

An exteriorized catheter, entering the dome of the UB, was placed at the back of the neck. A regimen of 400 μL of LPS purified by phenol extraction (1 μg/μL) (*E. coli* O55:B5; Sigma-Aldrich, St. Louis, MO, USA) was prepared to induce IC. Twice-weekly instillations of LPS via the exteriorized catheter for 3 weeks were used to induce a long-lasting and chronic injury to the urothelium. In the sham control group, LPS was replaced with 400 μL sterilized saline. The treatment solution was prepared with 15 mg dehydrated HACM extracts (HCT Regenerative Co. Ltd.; Taichung, Taiwan; the content of dehydrated HACM extracts was listed in Supplementary S1) in 7.5 mL normal saline. Each rat was infused with 300 μL solution intravesically (dehydrated HACM extracts: 3 mg/kg) at 48 h after day 0, 7 and 14 of IC induction. On day 21, rats were sacrificed, and tissues of UB were harvested for analysis. Before sacrificing the rats, urine samples of 24 h were collected using metabolic cages on the fourth day after IC induction to determine the color and blood content of the urine.

### 2.4. Histological Examinations

The UB were fixed in 4% paraformaldehyde on day 21 after the first LPS instillation. The UB of the rats in the sham control group were collected on day 7 after the first normal saline instillation. We froze the UB tissues, cut them on a cryostat at 5-μm thickness and then stained them with hematoxylin and eosin (H&E). In addition, cytokeratin immunostaining (Keratin, Pan Ab-1; Thermo Scientific, Foster City, CA, USA), toluidine blue staining (Toluidine blue-O; Daejung Chemicals and Metals, Seoul, Korea), and Masson’s trichrome staining (Junsei Chemical, Tokyo, Japan) were performed to evaluate the epithelial denudation, mast-cell infiltration, and tissue fibrosis, respectively. The slides would be transferred into digital ones using the NanoZoomer Digital Pathology system (Hamamatsu Photonics, Hamamatsu, Japan). In immune cells infiltration, we selected an area with the most densely clustered immunolabeled mast cells under low magnification. We had the results interpreted by an expert under the magnification of 200×. The counting process was similar to that while assessing cytokeratin and fibrosis. In mast cell infiltration, we also selected an area with the most densely clustered immunolabeled mast cells and had it read by an automated cell counter (cell/mm^2^).

### 2.5. Estimation of Concentrations of Inflammatory Cytokines, NF-κB Activity and Oxidative Markers

On day 21, the UB tissues were homogenized with 100 μL ice-cold tissue RIPA lysis buffer. The homogenate was incubated at 4 °C for 30 min and centrifuged at 12,000× *g* at 4 °C for 20 min. The supernatants were obtained as the total protein extracts. Total protein concentration was measured with the bicinchoninic acid (BCA) assay kit (Thermo Fisher Scientific, Waltham, MA, USA).

TNF-α, IL-6, IL-8, IL-1β, and NF-κB levels were measured in UB tissues homogenates using ELISA kits for rats (Biolegend, San Diego, CA, USA). The supernatants nuclear extracts were prepared using the NE-PER Nuclear and Cytoplasmic Extraction system (Thermo Fisher Scientific, Waltham, MA, USA). For each assay, 10 μg of nuclear extracts from UB tissues’ homogenates were used in a TransAM NF-κB p65 ELISA kit (Active Motif, Carlsbad, CA, USA). The level of malondialdehyde (MDA) was determined using the Lipid Peroxidation MDA assay kit (Colorimetric/Fluorometric) (Cat# ab118970, Abcam, Cambridge, MA, USA). Superoxide dismutase (SOD) and glutathione peroxidase (GSH-Px) activities were determined using the SOD Colorimetric activity kit (Cat# EIASODC, Thermo Fisher Scientific, Waltham, MA, USA) and GSH-Px ELISA kit (Cat#11352, Cusabio Biotech Co., Ltd., Wuhan, China), respectively. All steps were completed according to the manufacturer’s instructions.

### 2.6. Western Blotting

UB tissues were collected on day 21 and homogenized in 100 μL tissue RIPA lysis buffer for 30 min. Protein expression levels of caspase-3, Bax, and Bcl-2 were determined using Western blot analysis. In this study, the primary antibodies were anti-caspase-3, Bax, Bcl-2, and GAPDH (clone W17079A, BioLegend, San Diego, CA, USA). Each membrane was re-probed with an antibody against GAPDH, which functioned as an internal control for equalizing the protein loading. The band density was quantified with ImageJ software (Version 1.8.0, National Institute of Health, Bethesda, MD, USA).

### 2.7. Statistical Analysis

Experimental data are presented as the mean ± standard deviation (SD) and interquartile range (IQR). Comparisons among multiple treatments were performed using Tukey’s honest significant difference test after one-way ANOVA in GraphPad Prism (version 8; GraphPad Software, La Jolla, CA, USA). The α value of 5% was considered a cutoff for determining the statistical significance, and the actual statistical details were given at the Supplementary S2.

## 3. Results

### 3.1. Dehydrated HACM Extracts Improved Hematuria and Histological Pathologies in Rats with LPS-Induced IC

**Figure 1:**
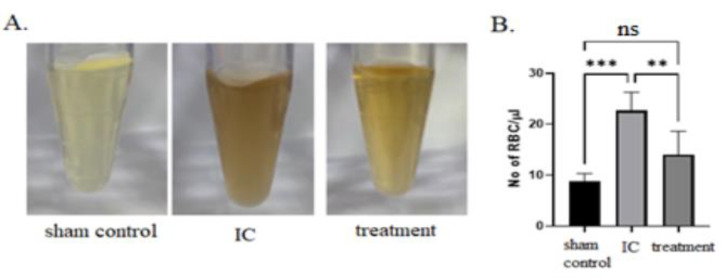
(**A**) General appearances of urine and (**B**) hematuria measurements. The hematuria was lessened after instilling dehydrated HACM extracts. ns: non-significant ** *p* < 0.01 *** *p* < 0.001.

**Figure 2:**
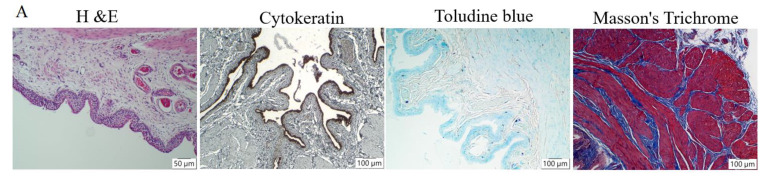

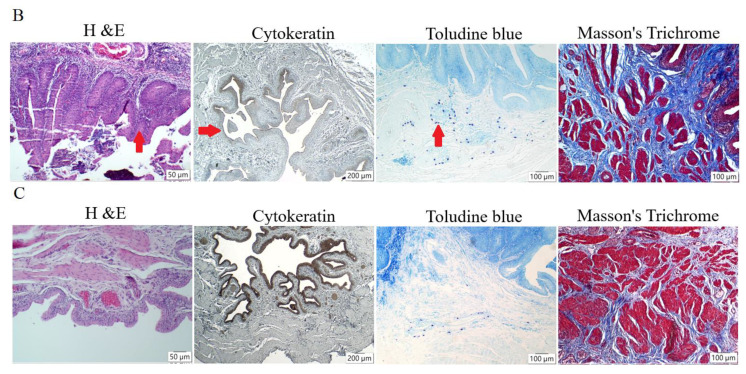
The comparative panoramic images among the three groups. (**A**) Histological presentation of the sham control group. (**B**) Histological pathologies of the IC group. (**C**) Histological changes in the treatment group. In H&E stains, rats with LPS-induced IC lost the normal architecture of urothelium and recovery was achieved after being treated with dehydrated HACM extracts. In cytokeratin and toluidine blue, rats treated with dehydrated HACM extracts showed recoveries of urothelial denudation and less mast cell infiltration. Masson’s trichrome showed less fibrosis in rats treated with dehydrated HACM extracts. Red arrow in (**B**) H&E: thick re-epithelialization; Red arrow in (**B**) cytokeratin: urothelial denudation; Red arrow in (**B**) Toludine blue: mast cell infiltration.

**Figure 3:**
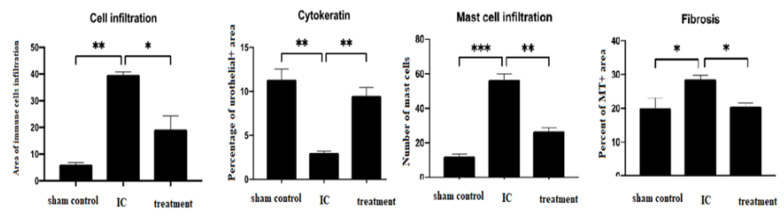
The measurements of the histological parameters. The figure indicates that dehydrated HACM extracts could lessen the thick re-epithelization of the urothelium, reconstruct the urothelial denudation, decrease mast cell infiltration, and ameliorate the fibrosis. * *p* < 0.05 ** *p* < 0.01 *** *p* < 0.001.

In general, appearances of urine samples, color, and hematuria were improved by dehydrated HACM extracts (Figure 1). In histological assessment, dehydrated HACM extracts could effectively improve the pathological changes in rats with LPS-induced IC. In hematoxylin and eosin (H&E) examination, the mixed inflammatory cells’ infiltration and abnormally thick re-epithelialization (Figure 2 and Figure 3) were observed in LPS-induced IC. LPS could also decrease cytokeratin-stained urothelium and increase toluidine blue-stained mast cell infiltration (Figure 2 and Figure 3). As shown by the results of Masson’s trichrome staining, tissue fibrosis was also enhanced in LPS-induced IC (Figure 2 and Figure 3). The abovementioned abnormalities were reversed in the treatment group by dehydrated HACM extracts (Figure 2 and Figure 3).

### 3.2. Dehydrated HACM Extracts Decreased Inflammatory Cytokines and Fibrosis-Related Encoding Genes in UB Tissues of Rats with LPS-Induced IC

**Figure 4:**
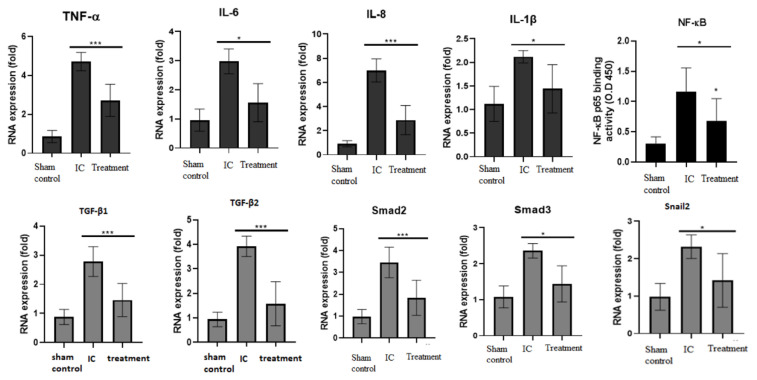
The Expressions of the inflammatory cytokines and fibrosis- related encoding genes. The figure indicates that dehydrated HACM extracts could down-regulate the inflammatory response and decrease fibrotic pathogenesis. * *p* < 0.05 *** *p* < 0.001.

The expressions of the inflammatory biomarkers TNF-α, IL-6, IL-1β, and NF-κB by ELISA in the UB tissues were the higher in the IC group compared to the treatment group and the sham control group. (Figure 4). Dehydrated HACM extracts could effectively down-regulate the fibrosis-related encoding genes of TGF-β1/2, smad family, and snail2 (Figure 4).

### 3.3. Dehydrated HACM Extracts Triggered Anti-Oxidative Effects in UB Tissues of Rats with LPS-Induced IC

**Figure 5:**
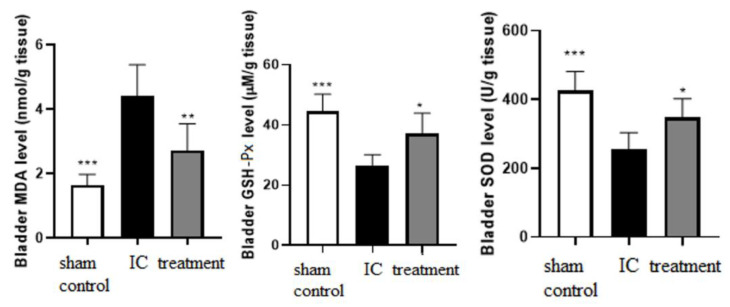
The oxidative stress biomarkers. The elevated MDA levels in the IC group could be suppressed after treatment with the dehydrated HACM extracts. Meanwhile, the suppressed GSH-Px and SOD levels in the IC group could be restored after instilling dehydrated HACM extracts. The figure indicates that dehydrated HACM extracts could reduce oxidative stress by decreasing polyunsaturated fatty acid peroxidation and increasing anti-oxidative enzymes. * *p* < 0.05 ** *p* < 0.01 *** *p* < 0.001.

We examined the levels of oxidative stress biomarkers, including MDA, GSH-Px, and SOD in UB homogenates. The MDA (Figure 5) concentrations in UB tissues were significantly elevated by LPS in the established IC model. However, the instillation of dehydrated HACM extracts could significantly reduce the elevated MDA in the UB tissues. In addition, dehydrated HACM extracts could restore the activities of the antioxidant enzyme GSH-Px and SOD (Figure 5) in the UB tissues that were decreased by LPS instillation.

### 3.4. Dehydrated HACM Extracts Might Enhance Anti-Apoptosis in Rats UB Tissues with LPS-Induced IC

**Figure 6:**
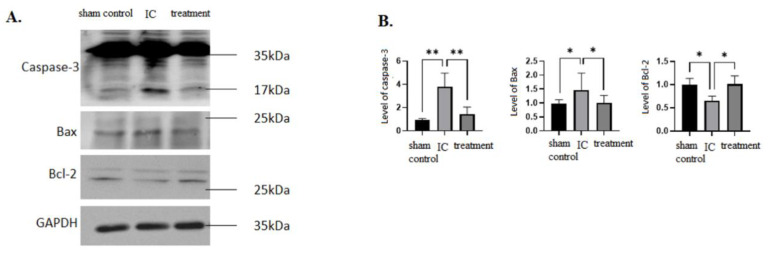
The pro-apoptotic and anti-apoptotic assays. (**A**,**B**) The expressions of caspase-3 and Bax were higher in the IC group than in the treatment group, while the anti-apoptotic Bcl-2 was higher in the treatment group than in the IC group. This figure indicates that dehydrated HACM extracts might suppress the pro-apoptotic ability and enhance the anti-apoptotic ability. * *p* < 0.05 ** *p* < 0.01.

The expressions of the pro-apoptotic proteins, caspase-3 and Bax, were significantly higher in the IC group than in the other two groups in Western blots (Figure 6). However, the index of anti-apoptotic protein, Bcl-2 was significantly higher in the treatment group compared to the IC group (Figure 6). These results suggested that the instillation of dehydrated HACM extracts could recover the elevated pro-apoptotic microenvironment in LPS-induced IC. Additionally, the down-regulated anti-apoptotic microenvironment in LPS-induced IC may be elevated upon instilling dehydrated HACM extracts.

## 4. Discussion

**Figure 7:**
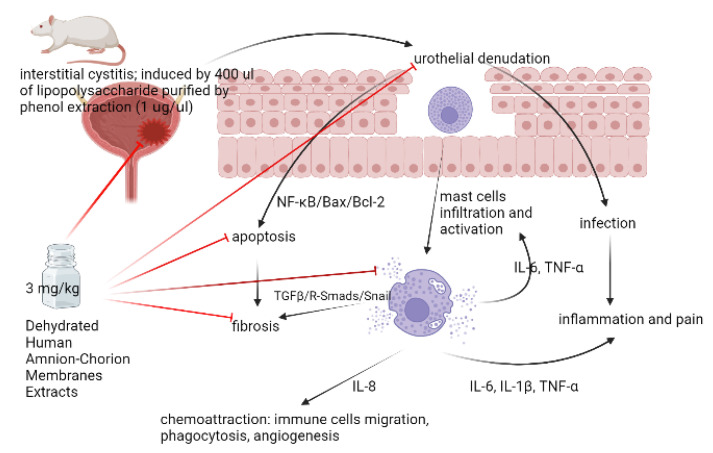
The possible mechanism of dehydrated HACM extracts in treating IC. In our study, the instillation of dehydrated HACM extracts could decrease the inflammatory responses, ameliorate the fibrotic reactions, and enhance the anti-apoptotic microenvironments in rats’ UB with LPS-induced IC.

In our experiment, we found that instilling dehydrated HACM extracts into the rats’ UB with LPS-induced IC could improve the pathological changes (Figure 7). Moreover, the inflammatory hyperplastic urothelium, the urothelial denudation, the fibrotic changes, and mast cell infiltrations were ameliorated. Although hematuria is not a vital sign in IC, approximately 40% diagnosed with IC would suffer from it [22]. In our model of LPS-induced IC, hematuria was significantly higher in the treatment group than in the sham control group. Hematuria, caused by LPS instillation, was also significantly improved after treatment with dehydrated HACM extracts. The inclusion of glomerulation into diagnostic criteria remains elusive; however, waterfall bleedings and Hunner’s lesions (HLs) are related. Nonetheless, there are no direct links between hematuria and life-threatening or symptomatic IC [22,23]. The former might be associated with smaller functional capacity of UB and urgency [24]. Since HLs were considered a sign of frequent recurrence [24], treating hematuria could be meaningful in preventing IC deterioration to some extent.

In the inflammatory cytokines and chemokines, we observed that dehydrated HACM extracts could lower IL-6 and TNF-α, which were essential to the development of mast cells [25], and further reduce the infiltrating mast cells in UB tissues. Moreover, in animal models [26], it was found that mast-cell infiltration could be related to inflammation and lower urinary tract symptoms. Although there were still disputes about the diagnostic role of mast cells in IC [27], mast-cell infiltration might be considered a feature differentiating IC from overactive bladder syndrome [28,29] in patients with HL. Furthermore, the counts of 32/mm^2^ would be the best cutoff [30,31]. In addition, it was found that patients with fewer mast-cell infiltration might have higher rates of treatment response to hydrodistention, and treatment effects would also exist longer [30]. Thus, being able to decrease mast-cell infiltration and related cytokines and chemokines, instilling dehydrated HACM extracts might have chance of relieving the lower urinary tract symptoms of patients in future human studies.

We also found that dehydrated HACM extracts could down-regulate NF-κB expression in inflammatory assessments. NF-κB triggered by either canonical or non-canonical pathways could arouse an inflammatory loop and is critical to the pathogenesis of IC [32,33]. In addition to its inflammatory effects, NF-κB plays a crucial function in regulating the cell cycle and cellular apoptosis. The pro- or anti-apoptotic effect of NF-κB was dependent on different cell lines [34]. In our IC model, the decreased anti-apoptotic Bcl-2 and enhanced pro-apoptotic Bax were observed, and the treatment of dehydrated HACM extracts reversed these two changes. This result suggested that NF-κB might possess a pro-apoptotic effect in IC pathogenesis via upregulating Bax and down-regulating Bcl-2. In addition, the dehydrated HACM extracts could restore the damaged tissues via repairing the dysregulated NF-κB/Bax/Bcl-2 pathway. Furthermore, the oxidative stress assessment revealed an increased MDA, decreased SOD, and decreased GSH-Px in the IC model. This imbalanced equilibrium would produce reactive oxygen species (ROS) and build the pro-apoptotic microenvironment of IC [35]. By down-regulating ROS, dehydrated HACM extracts could also ameliorate this pro-apoptotic microenvironment.

Another advanced pathogenesis of IC was fibrosis. It was an irreversible consequence of chronic inflammation and repeated injuries to the urothelium. In molecular aspects, fibrosis was a maladjusted condition of extracellular matrix (ECM), and the abnormal deposition of ECM would cause excessive scarring and weaken the structure of diseased tissues. The direct impact of fibrosis on patients with IC was to deteriorate their compliance with urinary bladders, worsening lower urinary tract symptoms, such as urgency and frequency. Moreover, some refractory situations might need surgical intervention [36,37], especially in patients with HLs. Herein, preventing the occurrence of fibrosis could be a hallmark in treating IC. In our IC model, we observed that the elevated TGFβ1/2 were accompanied by elevated Smad 2/3, presuming that one of the fibrotic mechanisms was the intracellular cascades of R-Smads’ phosphorylation via the signaling from TGFβ1/2. The upregulated Smad 2/3 would further stimulate the downstream pro-fibrotic factors. In our experiment, the activated TGFβ/R-Smads could be further accompanied by enhanced Snail2, suggesting the activated epithelial-to-mesenchymal transition (EMT) by the axis of TGFβ/R-Smads/Snail was critical to the fibrotic changes of IC. Besides this, suppressing TGFβ1 was another meaningful function in hindering fibrosis since it could directly convert inactive fibroblasts to ECM-secreting myofibroblasts [38]. In summary, dehydrated HACM extracts could lessen fibrosis of IC via down-regulating TGFβ isoforms and EMT from the axis of TGFβ/R-Smads/Snail.

In all phenotypes, HLs were the most discussed subtype of IC among the current studies, making patients and urologists feel frustrated. HLs, known as Hunner’s ulcer, represent the distinctive inflammatory patches and abnormal angiogenesis at the walls of the UB under cystoscopy. The pathological etiologies of IC with and without HLs could be distinct [39]. Mast cell infiltration and urothelial denudation are more typical in IC with HLs, [40], and treating them could significantly improve the symptoms of patients with IC [24]. In addition to the intravesical steroid injections into the lesions, electrocautery is currently the most popular surgical method. However, repeated sessions would lead to UB contracture and, consequently decrease the bladder’s maximal capacity, especially in those with low initial maximal UB capacities [40]. The instillation of dehydrated HACM extracts could improve urothelial denudation and mast-cell infiltration in our LPS-induced IC model. This implies that it could probably improve the abnormal symptoms of IC, especially those with HLs.

In this study, we observed that dehydrated HACM extracts possessed comprehensive capacities in regulating various cytokines and chemokines to lessen the pathogenesis of IC. Furthermore, the process of irreversible fibrosis could also be ameliorated. Platelet-rich plasma (PRP), such as dehydrated HACM extracts, is enriched with a variety of growth factors that can be used to regulate inflammatory cytokines and repair damaged epithelial tissue. According to recent research, intravesical instillation of PRP can repair the damaged urothelium in rats’ and rabbits’ IC models by lowering inflammatory cytokines and hematuria. In human trials, monthly intravesical injection for 4 months could significantly lessen the signs and symptoms and reduce inflammatory cytokines [41]. Thus, our preclinical study provided similar results as the intravesical PRP, and it offers us a reference in assessing its efficacy in future human trials. Monthly intravesical injection of a suspension of dehydrated HACM extracts for 4 months seems a feasible human trial protocol in assessing its application on human IC. The major limitation of this study was that alternative pathogenic pathways, such as the Wnt pathway, were not evaluated. However, we established the first animal model, demonstrating the efficacy of dehydrated human HACM extracts in treating IC. We intend to eventually adapt this to a phase-1 human study based on these findings and establish other paths for investigation.

## 5. Conclusions

In future, intravesical instillation of dehydrated HACM extracts may provide another alternative for treating IC. Our study revealed the down-regulation of inflammatory TNF-α, IL-6, IL-1β, and NF-κB, down-regulation of pro-fibrotic TGFβ/R-Smads/Snail pathway, down-regulation of fatty acid peroxidation product MDA, up-regulation of anti-oxidative enzymes GSH-Px and SOD, down-regulation of pro-apoptotic caspase-3 and Bax, and up-regulation of anti-apoptotic Bcl-2. In conclusion, dehydrated HACM extracts could be a powerful remedy in treating IC by reconstructing the damaged urothelium, reducing mast-cell infiltration and inflammatory reactions, and ameliorating fibrotic changes.

## Data Availability

The data supporting this study’s findings are available from the corresponding author upon reasonable request.

## Supplementary Materials

The following supporting information can be downloaded at: https://www.mdpi.com/article/10.3390/life12111693/s1, Supplementary S1. The main modulatory and stimulating factors contained in the dehydrated HACM extracts; Supplementary S2: The actual statistical information in every figure.
