# Supplementary material for: Dehydrated Human Amnion-Chorion Membrane Extracts Can Ameliorate Interstitial Cystitis in Rats by Down-Regulating Inflammatory Cytokines and Protein Coding Genes: A Preclinical Study
Source: Life (Basel). 2022 Oct 25;12(11):1693. doi: 10.3390/life12111693 (PMC9694506; doi:10.3390/life12111693)
Supplement: Supplementary file 1 [file life-12-01693-s001.zip › life-1822953-supplementary.pdf]

	Average concentration (pg/mg)
Growth factors	
Epidermal Growth Factors	4.88
Heparin Binding Epidermal Growth Factors	8.34
Fibroblast Growth Factors-4	23.73
Fibroblast Growth Factor-7	5.67
Vascular Endothelial Growth Factors	972.6
Growth Hormone	0.31
Insulin-Like Growth Factor Binding Protein-1	2534.8
Insulin-Like Growth Factor Binding Protein-2	6248.87
Insulin-Like Growth Factor Binding Protein-3	131268.96
Insulin-Like Growth Factor Binding Protein-4	78219.96
Insulin-Like Growth Factor Binding Protein-6	9739.38
Platelet-Derived Growth Factor AA	104.89
Platelet-Derived Growth Factor BB	59.21
Platelet-Derived Growth Factor AB	78.86
Transforming Growth Factor-α	0.03
Tissue Inhibitor of Metalloproteinase-1	3927.29
Tissue Inhibitor of Metalloproteinase-2	13431.53
Brain-derived Neurotrophic Factors	4.67
Inflammatory modulators	
Interleukin-1 Receptor Antagonist	25.36
Interleukin-1 Receptor Type I	127.64
Interleukin-6 Receptor Subunit α	130.40
Interleukin 12p40	18.70
IL-10 Receptor Binding Protein	39.54
Eotaxin	6.04
Eotaxin-2	16.19
Macrophage Inflammatory Protein-1α	70.81
Macrophage Inflammatory Protein-1β	55.17
Macrophage Inflammatory Protein-1δ	333.18
Regulated on Activation, Normal T-cell Expressed and Secreted	130.43
Chemokine Ligand-1	31.47
Tumor Necrosis Factor Receptor Type I	2723.18
Tumor Necrosis Factor Receptor Type II	2525.19

Supplementary S1. The main modulatory and stimulating factors contained in the dehydrated HACM extracts.

	Sham control group	IC group	Treatment group	p-value
Number of RBC [a] (n/μl)	9.20 ± 1.30<br>Median: 9.0<br>IQR [b] : 2.5	23.00 ± 3.94<br>Median: 23.0<br>IQR [b] : 6.0	14.20 ± 5.17<br>Median: 13.0<br>IQR [b] : 10.0	Sham control group *vs* IC group*:* <0.001***<br><br>IC group *vs* treatment group*:* 0.006**<br><br>Sham control group *vs* treatment group*:* 0.069

Supplementary S2

Supplementary S2.1. The actual value of Figure 1. All values were expressed with mean ± standard deviation, and median and interquartile range were provided. [a] red blood cell; [b] interquartile range ** p<0.01 ***p<0.001

	Sham control group	IC group	Treatment group	p-value
Area of immune cells infiltration (%)	8.60 ± 1.14<br>Median: 9.0<br>IQR [a] : 2.0	40.00 ± 1.58<br>Median: 40.0<br>IQR [a] : 3.0	20.60 ± 5.37<br>Median: 23.0<br>IQR [a] : 10.0	Sham control group *vs* IC group*:* 0.008**<br><br>IC group *vs* treatment group*:* 0.022*
Percent of urothelial+ area (%)	11.40 ± 1.67<br>Median: 11.0<br>IQR [a] : 3.0	3.00 ± 0.71<br>Median: 3.0<br>IQR [a] : 1.0	9.40 ± 1.52<br>Median: 9.0<br>IQR [a] : 2.0	Sham control group *vs* IC group*:* 0.008**<br><br>IC group *vs* treatment group*:* 0.008**
Number of mast cells (n/mm^2)	10.0 ± 1.0<br>Median: 10.0<br>IQR [a] : 2.0	57.80 ± 4.32<br>Median: 57.0<br>IQR [a] : 8.0	22.60 ± 2.30<br>Median: 23.0<br>IQR [a] : 4.0	Sham control group *vs* IC group*:* <0.001***<br><br>IC group *vs* treatment group*:* 0.008**
Percent of MT [b]+ area (%)	20.0 ± 5.0<br>Median: 21.0<br>IQR [a] : 9.5	27.20 ± 2.59<br>Median: 28.0<br>IQR [a] : 5.0	21.80 ± 1.79<br>Median: 23.0<br>IQR [a] : 3.0	Sham control group *vs* IC group*:* 0.032*<br><br>IC group *vs* treatment group*:* 0.035*

Supplementary S2.2. The actual numbers in Figure 3. All values were expressed with mean ± standard deviation, and median and interquartile range were provided. [a] interquartile range; [b] Masson's Trichrome; * p<0.05 ** p<0.01 ***p<0.001

	Sham control group	IC group	Treatment group	p-value
TNF-α: RNA expression (fold)	0.92 ± 0.29<br>Median: 0.9<br>IQR [a] : 0.5	4.90 ± 0.29<br>Median: 5.0<br>IQR [a] : 0.6	28.00 ± 0.39<br>Median: 28.0<br>IQR [a] : 0.7	IC group *vs* treatment group:<0.001***
IL-6: RNA expression (fold)	1.12 ± 0.37<br>Median: 1.2<br>IQR [a] : 0.7	0.30 ± 0.16<br>Median: 0.3<br>IQR [a] : 0.3	1.66 ± 0.50<br>Median: 1.6<br>IQR [a] : 1.0	IC group *vs* treatment group:0.024*
IL-8: RNA expression (fold)	0.84 ± 0.17<br>Median: 0.8<br>IQR [a] : 0.3	7.00 ± 2.13<br>Median: 7.3<br>IQR [a] : 3.7	2.88 ± 1.21<br>Median: 3.1<br>IQR [a] : 2.3	IC group *vs* treatment group: <0.001***
IL-1β: RNA expression (fold)	1.28 ± 0.16<br>Median: 1.2<br>IQR [a] : 0.30	2.12 ± 0.29<br>Median: 2.1<br>IQR [a] : 0.55	1.44 ± 0.52<br>Median: 1.5<br>IQR [a] : 0.9	IC group *vs* treatment group:0.033*
NF-κβ: p65 binding activity (OD450)	0.34 ± 0.09<br>Median: 0.3<br>IQR [a] : 0.2	1.23 ± 0.40<br>Median: 1.0<br>IQR [a] : 0.7	0.75 ± 0.37<br>Median: 0.7<br>IQR [a] : 0.7	IC group *vs* treatment group:0.043*
TGF-β1: RNA expression (fold)	0.94 ± 0.30<br>Median: 0.9<br>IQR [a] : 0.5	3.92 ± 0.94<br>Median: 3.9<br>IQR [a] : 1.7	1.58 ± 0.90<br>Median: 1.2<br>IQR [a] : 1.7	IC group *vs* treatment group: <0.001***
TGF-β2: RNA expression (fold)	0.88 ± 0.26<br>Median: 0.8<br>IQR [a] : 0.5	2.78 ± 1.15<br>Median: 2.7<br>IQR [a] : 2.10	1.46 ± 0.57<br>Median: 1.3<br>IQR [a] : 1.0	IC group *vs* treatment group: <0.001***
Smad2: RNA expression (fold)	0.98 ± 0.33<br>Median: 0.8<br>IQR [a] : 0.55	3.46 ± 1.56<br>Median: 3.2<br>IQR [a] : 2.75	1.84 ± 0.80<br>Median: 1.7<br>IQR [a] : 1.25	IC group *vs* treatment group: <0.001***
Smad3: RNA expression (fold)	1.08 ± 0.30<br>Median: 1.1<br>IQR [a] : 0.45	2.36 ± 0.46<br>Median: 2.3<br>IQR [a] : 0.75	1.44 ± 0.50<br>Median: 1.6<br>IQR [a] : 0.9	IC group *vs* treatment group:0.016*
Snail2: RNA expression (fold)	0.98 ± 0.36<br>Median: 0.9<br>IQR [a] : 0.7	2.32 ± 0.81<br>Median: 2.1<br>IQR [a] : 1.55	1.42 ± 0.72<br>Median: 1.4<br>IQR [a] : 1.2	IC group *vs* treatment group: 0.044*

Supplementary S2.3. The actual numbers in Figure 4. All values were expressed with mean ± standard deviation, and median and interquartile range were provided. [a] interquartile range. *p<0.05 ***p<0.001

	Sham control group	IC group	Treatment group	p-value
Bladder MDA level (nmol/ g)	1.64 ± 0.34 Median: 1.7 IQR [a] : 0.7	4.40 ± 0.98 Median: 4.3 IQR [a] : 1.9	2.72 ± 0.83 Median: 2.6 IQR [a] : 1.3	Sham control group *vs* IC group*:* <0.001*** IC group *vs* treatment group*:* 0.009**
Bladder GSH-Px level(uM/ g)	44.46 ± 5.76 Median: 43.2 IQR [a] : 11.4	26.62 ± 3.57 Median: 27.3 IQR [a] : 6.8	37.08 ± 6.84 Median: 33.9 IQR [a] : 1.9	Sham control group *vs* IC group*:* <0.001*** IC group *vs* treatment group*:*0.016*
Bladder SOD level (U/g)	425.92 ± 56.38 Median: 432.3 IQR [a] : 105.75	256.74 ± 47.35 Median: 239.30 IQR [a] : 89.2	347.52 ± 55.07 Median: 331.2 IQR [a] :108.2	Sham control group *vs* IC group*:* <0.001*** IC group *vs* treatment group*:*0.023*

Supplementary S2.4. The actual numbers in Figure 5. All values were expressed with mean ± standard deviation, and median and interquartile range were provided. [a] interquartile range. *p<0.05 **p<0.01 ***p<0.001

	Sham control group	IC group	Treatment group	p-value
Level of caspase 3	0.90 ± 0.16 Median: 0.9 IQR [a] : 0.3	4.50 ± 0.47 Median: 4.5 IQR [a] : 0.3	1.62 ± 0.23 Median: 1.6 IQR [a] : 0.4	Sham control group *vs* IC group*:* 0.006** IC group *vs* treatment group*:*0.008**
Level of Bax	0.94 ± 0.21 Median: 0.9 IQR [a]: 0.4	1.50 ± 0.42 Median: 1.4 IQR [a] : 03	1.00 ± 0.16 Median: 1.0 IQR [a] : 0.3	Sham control group vs IC group: 0.035* IC group vs treatment group: 0.038*
Level of Bcl-2	0.96 ± 0.21 Median: 1.0 IQR [a] : 0.4	0.66 ± 0.60 Median: 0.9 IQR [a] : 0.2	1.08 ± 0.16 Median: 1.0 IQR [a] : 0.3	Sham control group *vs* IC group*:* 0.032* IC group *vs* treatment group*:* 0.030*

Supplementary S2.5. The actual numbers in Figure 6. All values were expressed with mean ±

standard deviation, and median and interquartile range were provided. [a] interquartile range.
*p<0.05 **p<0.01
